# Incentive-Aware End-to-End Covert Routing for Space–Air–Ground Integrated Networks

**DOI:** 10.3390/s26154924

**Published:** 2026-08-04

**Authors:** Zhao Deng, Mingze Li, Nannan Sun, Shouxin Cao, Yue Gao, Yang Xu

**Affiliations:** 1 No. 30 Research Institute of China Electronics Technology Group Corporation, Chengdu 610041, China; dengz2694@cetcsc.com; 2 School of Computer Science and Technology, Xidian University, Xi’an 710071, China; limingze@stu.xidian.edu.cn (M.L.); nannansun@stu.xidian.edu.cn (N.S.); shouxin.cao@stu.xidian.edu.cn (S.C.); ygao@xidian.edu.cn (Y.G.); 3 China Satellite Network Application Co., Ltd., Beijing 100160, China

**Keywords:** covert communication, space–air–ground integrated networks, incentive-aware routing, zero-determinant strategy

## Abstract

Covert communication has emerged as a promising technique for protecting wireless transmissions by concealing the existence of legitimate communication from malicious wardens. However, achieving end-to-end covert communication in space–air–ground integrated networks (SAGINs) is challenging due to the coupled effects of satellite-to-ground relay access, ground multi-hop forwarding, and cooperative jamming. In this paper, we propose an incentive-aware end-to-end covert routing framework for SAGINs, where a low Earth orbit (LEO) satellite delivers information to a ground destination through a selected relay base station and a self-organizing ground route. We first establish a two-stage SAGIN model and characterize the satellite-to-ground covert capacity under satellite sidelobe interference, as well as the ground-route covert performance in the presence of multiple wardens and cooperative jammers. Since jammers are self-interested and incur power costs when generating artificial interference, we design an incentive mechanism to stimulate cooperative jamming for enhancing ground-route covertness. Specifically, the reward allocation and jamming-power response are jointly derived by considering both the route-dependent covertness gain and the power cost of jammers. Based on the resulting route-dependent utility, the ground routing problem is further transformed into a shortest-weighted path-finding problem. To improve the long-term stability of satellite-to-ground relay access, we model the repeated interaction between the LEO satellite transmitter and the satellite warden as a base-station selection process and develop a zero-determinant strategy to stabilize the long-term expected utility relation under different warden monitoring policies. Simulation results demonstrate that the proposed framework effectively balances satellite-to-ground covert capacity and ground-route utility, outperforms baseline relay selection schemes, and achieves stable long-term covert routing performance against uncertain warden behaviors.

## 1. Introduction

Space–air–ground integrated networks (SAGINs) are emerging as a key architecture for next-generation wireless systems, where low Earth orbit (LEO) satellites, aerial platforms, and terrestrial infrastructures are integrated to provide ubiquitous and resilient connectivity [1]. By extending communication services beyond the coverage limitation of conventional terrestrial networks, SAGINs are expected to support wide-area Internet-of-Things (IoT) access, emergency communication, remote sensing, and mission-critical information delivery [2]. However, the open broadcast nature of wireless propagation also exposes SAGINs to severe security and privacy risks [3]. In particular, both satellite-to-ground links and ground multi-hop links can be monitored by malicious wardens, who may attempt to detect the existence of confidential communication before launching further attacks. Therefore, ensuring communication security in SAGINs requires not only protecting the transmitted content, but also concealing the occurrence of transmission itself [4].

Recent studies have further demonstrated the importance of satellite networks and secure wireless communications in future integrated network architectures. For integrated satellite–terrestrial networks, the performance analysis of partial non-orthogonal multiple access under co-channel interference has revealed the close coupling between satellite and terrestrial transmissions, highlighting the importance of effective interference management in heterogeneous network environments [5]. Secure communication studies involving aerial platforms have also shown that properly designed jamming beamforming can be exploited to protect legitimate transmissions against eavesdropping threats [6]. These advances underscore the important roles of satellite communications, interference management and utilization, and security protection in future integrated wireless networks.

Covert communication, also known as low probability of detection (LPD) communication, has been recognized as an effective technique for hiding legitimate transmissions from wardens [7,8]. Different from conventional encryption-based methods, which mainly protect message content, covert communication aims to prevent adversaries from reliably determining whether a transmission is taking place. The warden’s detection performance is commonly characterized by the false alarm probability and the missed detection probability. The fundamental limit of covert communication was first studied in [9], where the square-root law was established. Since then, extensive research efforts have investigated covert communication over additive white Gaussian noise (AWGN) channels [10,11], secrecy-aware wireless systems [12], ambient backscatter systems [13], and intelligent reflecting surface (IRS)-assisted systems [14,15]. Among various enabling techniques, cooperative jamming has been widely adopted to confuse wardens by injecting artificial interference, thereby improving covert communication performance.

Despite these advances, most existing studies focus on link-level covert communication, such as point-to-point links, relay-assisted links, or single-hop systems. Such settings are insufficient for SAGINs, where information delivery from a LEO satellite to a ground destination generally involves a two-stage process: the satellite first selects a relay base station through the satellite-to-ground link, and the selected base station then forwards the message through a self-organizing ground multi-hop route. This introduces a coupled end-to-end covert routing problem. On the one hand, the satellite-to-ground covert capacity depends on the antenna gains, satellite sidelobe interference, propagation distance, and the satellite warden’s monitoring position. On the other hand, the ground-route covertness is affected by the selected relay nodes, the spatial distribution of ground wardens, and the jamming power supplied near the wardens. Therefore, selecting a relay base station based only on the satellite-to-ground link or optimizing only the ground route may lead to suboptimal end-to-end covert performance.

Another important issue lies in the practical deployment of cooperative jammers. Existing jamming-assisted covert communication studies often assume that jammers are fully cooperative and willing to generate artificial interference without compensation. However, in a distributed SAGIN-assisted ground network, jammers may be independent and self-interested devices. Since generating jamming signals consumes their own power resources, they may not voluntarily participate in covertness enhancement unless appropriate incentives are provided. Moreover, the routing decision itself is affected by the incentive cost: a route that appears covert may become inefficient if maintaining its covertness requires excessive reward expenditure. Hence, an effective end-to-end covert routing design should jointly account for route-dependent covertness gain and the cost of incentivizing cooperative jamming.

Furthermore, SAGIN covert routing is not only a one-shot decision problem. During long-term communication, the LEO satellite can dynamically switch among candidate base stations to reduce the opportunity for the satellite warden to continuously approach and monitor the actual relay base station. However, the warden may also adapt its monitoring target across communication cycles. This repeated interaction makes long-term relay access stability an important concern. A desirable routing framework should therefore provide not only instantaneous end-to-end covert utility, but also a mechanism to stabilize long-term satellite-to-ground relay selection under uncertain warden monitoring behaviors.

Motivated by the above observations, this paper investigates incentive-aware end-to-end covert routing for SAGINs. We consider a two-stage SAGIN communication model in which a LEO satellite transmits information to a ground destination through a selected relay base station and a self-organizing ground route. The overall routing utility jointly incorporates the satellite-to-ground covert capacity and the ground-route utility. For the ground segment, self-interested jammers are incentivized to generate artificial interference for protecting the multi-hop route. Based on the derived route-dependent utility, the ground routing problem is converted into a shortest-weighted path-finding problem. For the satellite-to-ground segment, a zero-determinant (ZD) strategy is further introduced to stabilize the long-term utility relation in the repeated base-station selection process.

Current SAGIN-oriented covert communication schemes mainly focus on physical-layer transmission designs, such as beamforming, coverage optimization, or single-link covert capacity, without considering incentive-aware ground multi-hop routing. Existing multi-hop covert routing and incentive-routing studies are mainly developed for terrestrial networks and do not account for satellite-to-ground relay access or the repeated interaction between the satellite transmitter and the warden. Moreover, existing SAGIN routing studies generally do not jointly consider covert transmission, self-interested cooperative jammers, and long-term utility stabilization. Therefore, the key novelty of the proposed framework lies in coupling incentive-aware ground-route formation with ZD-based long-term relay selection through a unified end-to-end utility, thereby connecting short-term distributed cooperation with long-term strategic stability.

The main contributions of this paper are summarized as follows:

- End-to-end covert routing framework for SAGINs: We develop an incentive-aware covert routing framework for SAGINs by jointly considering satellite-to-ground relay access and ground multi-hop forwarding. Different from conventional link-level covert communication studies, the proposed framework evaluates the end-to-end routing utility by incorporating both the satellite-to-ground covert capacity and the optimized ground-route utility.
- Incentive-aware ground routing with self-interested jammers: We characterize the ground-route covert performance in the presence of multiple wardens and cooperative jammers. Considering that jammers incur power costs when generating artificial interference, we design an incentive mechanism to determine the reward allocation and jamming-power response. The resulting route-dependent utility enables the ground routing problem to be transformed into a shortest-weighted path-finding problem, which can be efficiently solved by classical routing algorithms.
- Long-term relay access stabilization via ZD strategy: We model the long-term interaction between the LEO satellite transmitter and the satellite warden as a repeated base-station selection process. A ZD-based strategy is developed to stabilize the long-term expected utility relation under different warden monitoring policies, thereby improving the predictability and robustness of satellite-to-ground relay access.
- Comprehensive performance evaluation: We conduct numerical simulations to evaluate the proposed framework. The results demonstrate the necessity of jointly considering satellite-to-ground covert capacity and ground-route utility, verify the impact of key satellite and ground network parameters, and show that the ZD strategy can stabilize long-term performance under uncertain warden behaviors.

The remainder of this paper is organized as follows. Section 2 reviews related work. Section 3 presents the SAGIN system model and evaluates the covert performance of both the satellite-to-ground link and the ground routing process. Section 4 develops the incentive-aware ground routing design and transforms the routing problem into a shortest-weighted path-finding problem. Section 5 introduces the ZD-based long-term base-station selection strategy. Section 6 provides simulation results. Finally, Section 7 concludes this paper.

## 2. Related Work

Covert communication aims to conceal the existence of legitimate transmissions by limiting the detection capability of potential wardens. Bash et al. [9] established the square-root law for covert communication over additive white Gaussian noise channels, while Wang et al. [10] further characterized its fundamental information-theoretic limits. Subsequent studies extended covert transmission to multiple-input multiple-output systems and other complex wireless environments [16]. More recently, physical-layer covert communication has been investigated in satellite-ground integrated sensor networks and near-field integrated sensing and communication systems. Specifically, Ni et al. [17] developed an FH-DL-MPWFRFT-based scheme to enhance waveform obfuscation and interception resistance in satellite-ground links. Xu and Ji [18] introduced a vulnerable region control framework to jointly guarantee sensing accuracy, user quality of service, and communication covertness in near-field ISAC systems. Beyond waveform-level and single-stage transmission designs, subsequent studies have increasingly explored artificial jamming as an effective means of further enhancing covert communication performance.

Artificial jamming increases the uncertainty of the received signal at a warden and thereby enhances covert communication performance. Sobers et al. [19] showed that an uninformed jammer can substantially improve communication covertness, while Shahzad et al. [20] exploited a full-duplex receiver to generate randomized artificial interference. Zheng et al. [21] investigated distributed cooperative jamming, and He et al. [22] jointly optimized jammer selection and power control. For aerial communication scenarios, Gu et al. [23] proposed a dual unmanned aerial vehicles (UAV) architecture with dual-mode stochastic jamming and jointly optimized the UAV trajectories, velocities, accelerations, and transmit powers to maximize the average covert rate. These studies verify the effectiveness of cooperative jamming, but they mainly optimize jamming strategies over predetermined links and rarely capture the coupling between jamming resource allocation and end-to-end route selection. In addition to power control and artificial jamming, index modulation (IM) provides an energy- and spectrum-efficient transmission paradigm by embedding information into the indices of transmission entities, such as subcarriers, time slots, codes, and signal-generation parameters [24,25]. Its integration with covert communications may offer a complementary means of improving transmission efficiency and reducing the detectability of legitimate signals without necessarily increasing the transmit power.

Multi-hop transmission improves communication covertness by decomposing a long-distance link into several shorter hops, thereby reducing the signal exposure and detection risk of each transmission. Lv et al. [26] investigated covert communication assisted by a multi-antenna relay, while Peng and Soljanin [27] studied covert and low-delay coded message delivery in mobile relay networks. Sheikholeslami et al. [28] formulated the multi-hop covert routing problem in the presence of multiple collaborating wardens and developed routing algorithms for throughput maximization and end-to-end delay minimization. Wang et al. [29] further jointly optimized the hop count, coding rate, and transmit power of each hop against UAV surveillance. These studies demonstrate the covert advantages of multi-hop forwarding; however, they primarily determine routes according to physical layer covertness metrics and generally assume that relay and jamming resources remain continuously available.

In decentralized wireless networks, relay and jamming nodes may behave selfishly because packet forwarding and artificial interference generation consume their local resources. Buttyán and Hubaux [30] introduced a virtual currency mechanism to stimulate node cooperation, while Zhong et al. [31] developed a credit-based mechanism for self-organizing networks. Stanojev et al. [32] employed spectrum leasing to incentivize distributed cooperative transmission. More closely related to covert routing, Xie et al. [33] incorporated node cooperation costs into route utility and developed an incentive-aware routing framework for covert communication in decentralized multi-hop networks. Existing incentive-based covert routing studies mainly address terrestrial networks and do not jointly consider satellite-to-ground covert access, ground multi-hop route selection, route-dependent jamming power allocation, and jammer compensation. These limitations motivate the development of an end-to-end incentive-aware covert routing framework for SAGINs.

Recent multi-agent reinforcement learning (MARL) approaches provide another promising means of coordinating multiple distributed communication entities. For example, Miuccio et al. [34] developed a partially observable Markov game for reliable and energy-efficient MAC protocol learning, in which each device learns an individual policy based on local observations, while the base station broadcasts resource-level feedback for constructing feasible local rewards. Such a design demonstrates the potential of MARL to coordinate interacting entities under partial observability and heterogeneous operating conditions. In a dynamic SAGIN, similar learning mechanisms could be applied to adapt base-station selection, routing, and cooperative-jamming decisions when the topology, energy availability, and warden behavior are not fully known. Compared with MARL, the analytical game-theoretic approach adopted in this work provides explicit equilibrium solutions, stronger interpretability, and lower online computational overhead, but relies more strongly on accurate system and payoff models. Therefore, a hybrid design in which the analytical solution provides structural guidance or feasible constraints for MARL-based online adaptation represents a promising future direction.

## 3. System Model and Performance Evaluation

### 3.1. SAGIN Model

**Figure 1:**
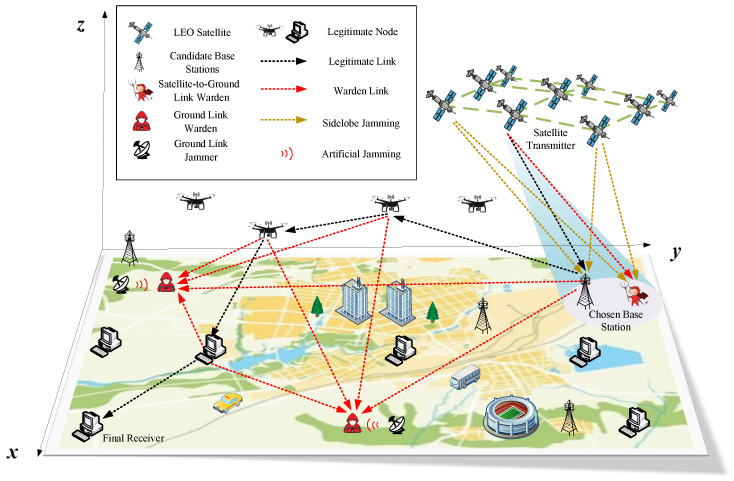
System model.

As illustrated in Figure 1, we consider a typical SAGIN model consisting of two communication phases: (1) the satellite-to-ground base station link, and (2) the self-organizing routing from the selected base station to an arbitrary ground destination node. The satellite-to-ground link mainly involves base station selection, and its covert capacity is denoted as $C_{L}$. The ground routing phase involves multi-hop relay node selection, including UAVs and ground relay nodes, as well as the configuration of jammer parameters. The covert performance of the ground routing phase is characterized by the base station utility $U_{B}$.

The covert capacity $C_{L}$ depends on satellite sidelobe interference and is jointly constrained by the detection and outage probability thresholds. The ground utility $U_{B}$ incorporates the power compensation required for external jammers. Accordingly, the overall utility of the satellite transmitter is defined as

(1) $$U_{T}=U_{B}\cdot \tanh\left(\eta C_{L}\right).$$

where η>0 is a scaling parameter used to balance the satellite-to-ground covert capacity and the ground-route utility. The hyperbolic tangent function provides a bounded and smooth mapping of $C_{L}$, thereby enabling joint optimization of base station selection and ground routing performance through protocol design.

In the satellite-to-base station link, information is transmitted from a satellite in the LEO satellite system, denoted by $L_{T}$. The satellite steers its signal beam toward a ground relay base station, which then forwards the received information to other specific locations. Let $B=\{B_{i}|i=1,2,\ldots ,N_{b}\}$ denote the set of candidate ground base stations, where $N_{b}$ is the number of candidate base stations. During transmission, the satellite signal may also cover a ground-based LEO satellite warden, denoted by $W_{L}$, which is assumed to be equipped with a corresponding satellite receiving antenna. This warden attempts to detect and intercept the transmitted information in the vicinity of the relay base station.

The ground network is modeled as a distributed self-organizing wireless network composed of multiple freely deployed legitimate nodes, including UAVs and ground relay nodes. All legitimate nodes are assumed to transmit signals with a fixed power *P*. Meanwhile, the ground link also contains wardens that observe ground transmissions. Unlike the satellite warden, which is unique and constrained by antenna configuration and specific base station coverage, ground wardens are assumed to be multiple and capable of joint analysis. Their deployment is more flexible, allowing them to be placed at key locations in the ground network. Let *M* denote the number of ground wardens, and let $\left\{W_{i}|i=1,2,\ldots ,M\right\}$ denote the set of ground wardens. The locations or channel conditions of potential ground wardens are assumed to be approximately available to the legitimate network. This information does not necessarily rely on real-time detection of a fully passive warden, but may be inferred from external monitoring infrastructure, prior security intelligence, historical observations, or risk assessments of predefined high-risk locations. Therefore, the proposed framework can also be viewed as a proactive protection mechanism against potential monitoring activities in such areas. The estimated information is assumed to remain sufficiently accurate within each route-selection interval.

To protect the covertness of ground communications, the base station recruits the same number of jammers, denoted by $J=\{J_{i}|i=1,2,\ldots ,M\}$, and places each jammer $J_{i}$ near the corresponding warden $W_{i}$. Let *D* denote the destination node. An arbitrary route from the selected base station *B* to *D* is denoted by $\Pi =\langle l_{1},l_{2},\ldots ,l_{K}\rangle$, where each link $l_{k}$ connects two intermediate nodes $S_{k}$ and $D_{k}$. The implementation details of covert-warden detection and localization, jammer discovery and recruitment, and secure dissemination of the required location and channel information are outside the scope of this study. This work focuses on incentive-aware jammer participation and end-to-end route optimization given the available estimates.

### 3.2. Satellite-to-Ground Link Covert Capacity

**Figure 2:**
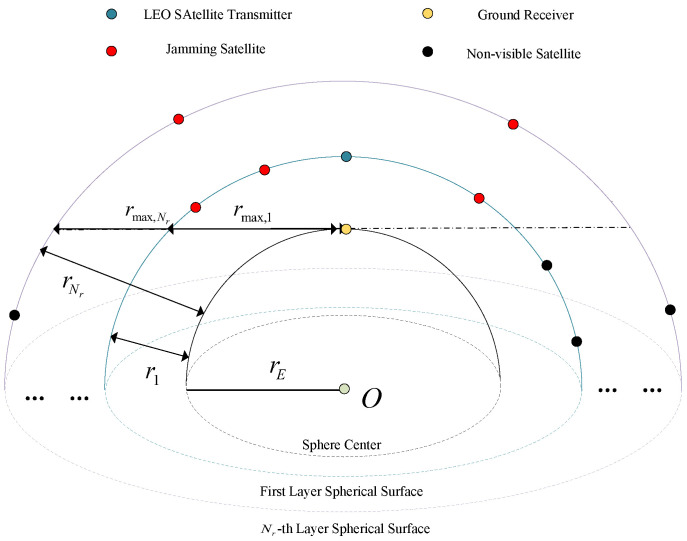
Satellite model.

We adopt the LEO satellite system model from [35]. As shown in Figure 2, this system consists of *N* satellites, each with transmit power $P_{L}$. The satellite signals are considered to follow a Gaussian distribution, i.e., $X_{L}∼N\left(0,1\right)$. The received signals at the ground base station and satellite warden can be expressed as

(2) $$\begin{array}{cc} Y_{B} & =\sqrt{G_{L}G_{B}P_{L}/r_{LB}^{\alpha }}\;h_{LB}X_{L}+Z_{B}+{\tilde{I}}_{B}, \end{array}$$

(3) $$\begin{array}{cc} Y_{W_{L}} & =\sqrt{G_{L}G_{W}P_{L}/r_{LW}^{\alpha }}\;h_{LW}X_{L}+Z_{W}+{\tilde{I}}_{W}, \end{array}$$

where:

- $G_{L}$ represents the transmitter’s antenna gain;
- $G_{B}$, $G_{W}$ represent the receiving antenna gains of the base station and warden respectively;
- $h_{LB}$, $h_{LW}$ represent the channel gains from transmitter to base station and warden respectively;
- $r_{LB}$, $r_{LW}$ represent the distances from transmitter to base station and warden respectively;
- α represents the path loss exponent for large-scale fading;
- $Z_{B}∼N\left(0,\sigma_{B}^{2}\right)$, $Z_{W}∼N\left(0,\sigma_{W}^{2}\right)$ represent the channel noise at the base station and warden;
- ${\tilde{I}}_{B}$ and ${\tilde{I}}_{W}$ denote the aggregate interference waveforms received at the base station and the warden, respectively. Their corresponding aggregate interference powers are denoted by $I_{B}$ and $I_{W}$.

The satellites are assumed to be uniformly distributed over $N_{r}$ orbital planes. The distance from each orbital plane to the center of the sphere is $r_{E}+r_{i}$, where $i\in \{1,2,\ldots ,N_{r}\}$. Here, $r_{E}$ denotes the Earth’s radius, and $r_{i}$ denotes the altitude of the *i*-th satellite’s spherical surface above the horizon. Therefore, each spherical surface contains $N_{s}=N/N_{r}$ uniformly distributed satellites, which follow a binomial point process (BPP). Since both the base station and the warden can be regarded as satellite signal receivers, we first focus on the base station and derive the aggregate interference generated by the satellite system.

Only satellites located above the base station’s horizon can generate co-channel interference through sidelobe leakage; these satellites are therefore referred to as interfering satellites. For the *i*-th spherical surface, the maximum distance $r_{max,i}$ between an interfering satellite and the base station is determined by the visible region. From Figure 2, we have $r_{max,i}=\sqrt{r_{i}^{2}+2r_{E}r_{i}}$. Let $N_{i}$ denote the number of interfering satellites on the *i*-th spherical surface, namely, the satellites whose distances to the base station satisfy $r_{i}\leq r\leq r_{max,i}$. The aggregate interference power received at the base station from all interfering satellites is expressed as

(4) $$I_{B}=\sum_{i=1}^{N_{r}}\sum_{j=1}^{N_{i}}G_{L,side}G_{B,side}r_{ij}^{-\alpha }P_{L}{\left|h_{ij}\right|}^{2},$$

where:

- $r_{ij}$ is the distance from the *j*-th interfering satellite ($j\in \{1,2,\ldots ,N_{i}\}$) on the *i*-th spherical surface ($i\in \{1,2,\ldots ,N_{r}\}$) to the base station;
- $G_{L,side}$ and $G_{B,side}$ are the sidelobe gains of the satellite transmitting antenna and base station receiving antenna, respectively;
- $h_{ij}$ represents the channel gain from the satellite to the base station, following a Rayleigh distribution with scale parameter σ;
- α denotes the path-loss exponent. Considering the predominantly line-of-sight propagation of LEO satellite-to-ground links, its typical value is set to α=2 in this paper.

To determine the effective number of interfering satellites, we calculate the ratio between the spherical cap area defined by the base station’s horizon and the total surface area of the *i*-th spherical surface. This yields the cumulative distribution function (CDF) of the distance between a satellite on the *i*-th spherical surface and the base station as

(5) $$F_{i}\left(r\right)=\frac{r^{2}-r_{i}^{2}}{4r_{E}\left(r_{E}+r_{i}\right)},\;r_{i}\leq r\leq 2r_{E}+r_{i}.$$

The interfering satellites are those satisfying $r_{i}\leq r_{ij}\leq r_{max,i}$. Let $p_{i}=\frac{r_{i}}{2(r_{E}+r_{i})}$ denote the probability that a satellite on the *i*-th spherical layer is visible to the considered ground receiver. Since each layer contains $N_{s}=N/N_{r}$ satellites, the number of visible interfering satellites, denoted as $N_{i}$, follows $N_{i}∼\mathrm{Binomial}\left(N_{s},p_{i}\right)$. Consequently, we have

(6) $$E\left[N_{i}\right]=\frac{N}{N_{r}}\frac{r_{i}}{2(r_{E}+r_{i})},\;\mathrm{Var}\left(N_{i}\right)=\frac{N}{N_{r}}\frac{r_{i}\left(2r_{E}+r_{i}\right)}{4{\left(r_{E}+r_{i}\right)}^{2}}.$$

The power distribution of the aggregate interference can be approximated by a Gamma distribution with shape parameter $k_{r}$ and scale parameter $\theta_{r}$, where the corresponding parameters are estimated using the method of moments. Specifically, the first and second raw moments of $r_{i,j}^{-2}$ and $|h_{i,j}{|}^{2}$ are given by

(7) $$E\left[r_{i,j}^{-2}\right]=\frac{1}{2r_{E}r_{i}}\ln\left(\frac{2r_{E}}{r_{i}}+1\right),\;E\left[r_{i,j}^{-4}\right]=\frac{1}{r_{i}^{3}\left(2r_{E}+r_{i}\right)},$$

(8) $$E\left[|h_{i,j}{|}^{2}\right]=2\sigma^{2},\;E\left[|h_{i,j}{|}^{4}\right]=8\sigma^{4}.$$

Based on these moments, we further derive the expectation and variance of the aggregate interference received at the base station. For notational convenience, define $A_{B}=G_{L,\mathrm{side}}G_{B,\mathrm{side}}P_{L}$. The interference contribution of the *j*-th visible satellite on the *i*-th spherical layer is denoted by $X_{i,j}=A_{B}r_{i,j}^{-2}{\left|h_{i,j}\right|}^{2}$. Since $r_{i,j}$ and $h_{i,j}$ are independent, the first and second raw moments of $X_{i,j}$ are given by

$$\mu_{i}≜E\left[X_{i,j}\right]=A_{B}E\left[r_{i,j}^{-2}\right]E[|h_{i,j}{|}^{2}]=\frac{A_{B}\sigma^{2}}{r_{E}r_{i}}\ln\left(\frac{2r_{E}}{r_{i}}+1\right),$$

$$\nu_{i}≜E\left[X_{i,j}^{2}\right]=A_{B}^{2}E\left[r_{i,j}^{-4}\right]E[|h_{i,j}{|}^{4}]=\frac{8A_{B}^{2}\sigma^{4}}{r_{i}^{3}\left(2r_{E}+r_{i}\right)}.$$

Conditioned on $N_{i}$, the interference contributions from the visible satellites on the *i*-th layer are independently and identically distributed. Therefore, by applying the expectation and variance formulas for a random sum, the mean aggregate interference power is obtained as

(9) $${\bar{I}}_{B}=k_{B}\theta_{B}=\sum_{i=1}^{N_{r}}E\left[N_{i}\right]\mu_{i}=A_{B}\sigma^{2}\sum_{i=1}^{N_{r}}\frac{E[N_{i}]}{r_{E}r_{i}}\ln\left(\frac{2r_{E}}{r_{i}}+1\right).$$

Similarly, the variance of the aggregate interference power is given by

(10) $$\begin{array}{cc} \sigma_{I,B}^{2} & =k_{B}\theta_{B}^{2}=\sum_{i=1}^{N_{r}}\left[E\left[N_{i}\right]\left(\nu_{i}-\mu_{i}^{2}\right)+\mathrm{Var}\left(N_{i}\right)\mu_{i}^{2}\right]\\ \; & =A_{B}^{2}\sigma^{4}\sum_{i=1}^{N_{r}}\left[\frac{8E[N_{i}]}{r_{i}^{3}\left(2r_{E}+r_{i}\right)}+\left(\mathrm{Var}\left(N_{i}\right)-E\left[N_{i}\right]\right)\frac{\ln^{2}\left(\frac{2r_{E}}{r_{i}}+1\right)}{r_{E}^{2}r_{i}^{2}}\right]. \end{array}$$

Accordingly, the shape and scale parameters of the moment-matched Gamma distribution are expressed as

$$k_{B}=\frac{{\bar{I}}_{B}^{\;2}}{\sigma_{I,B}^{2}},\;\theta_{B}=\frac{\sigma_{I,B}^{2}}{{\bar{I}}_{B}}.$$

The mean and variance of the aggregate interference received by the warden, denoted by ${\bar{I}}_{W}$ and $\sigma_{I,W}^{2}$, respectively, can be derived in the same manner by replacing the base-station-side antenna gain and distance-related statistics with their corresponding warden-side counterparts.

To characterize the applicability and approximation error of the Gaussian model in a multi-layer LEO constellation, let $Y_{i,j}$ denote the interference contribution of the *j*-th visible interfering satellite on the *i*-th spherical layer, i.e., $Y_{i,j}=G_{L,side}G_{B,side}P_{L}r_{ij}^{-\alpha }{\left|h_{ij}\right|}^{2}$, and then the aggregate interference can then be expressed as $I_{B}=\sum_{i=1}^{N_{r}}\sum_{j=1}^{N_{i}}Y_{i,j}$. Define $\mu_{i}=E\left[Y_{i,j}\right]$, $\sigma_{i}^{2}=Var\left(Y_{i,j}\right)$, and $\rho_{i}=E[|Y_{i,j}-\mu_{i}{|}^{3}]$. Under the independent satellite-location and fading assumptions, all third absolute moments are finite. According to the Berry–Esseen theorem, the Kolmogorov distance between the standardized aggregate interference and a standard Gaussian random variable satisfies

(11) $$\epsilon_{G}≜\underset{x}{\sup}\left|\mathrm{Pr}\left(\frac{I_{B}-{\bar{I}}_{B}}{\sigma_{I,B}}\leq x\right)-\Phi \left(x\right)\right|\leq C_{\mathrm{BE}}\frac{\sum_{i=1}^{N_{r}}N_{s}\rho_{i}}{{\left(\sum_{i=1}^{N_{r}}N_{s}\sigma_{i}^{2}\right)}^{3/2}}=C_{\mathrm{BE}}\sqrt{\frac{N_{r}}{N}}\frac{\sum_{i=1}^{N_{r}}\rho_{i}}{{\left(\sum_{i=1}^{N_{r}}\sigma_{i}^{2}\right)}^{3/2}}.$$

where $C_{BE}$ is a universal constant and Φ(·) denotes the standard Gaussian CDF. This bound shows that the approximation accuracy depends jointly on *N*, $N_{r}$, and the heterogeneity of the layer-dependent interference moments. When the moments of different layers remain bounded and no individual satellite or layer dominates the total interference variance, $\epsilon_{G}$ decreases at the rate $O(N^{-1/2})$. Thus, the approximation becomes increasingly accurate as the effective number of interfering satellites increases. For a fixed *N*, increasing $N_{r}$ does not necessarily degrade the approximation when the interference contributions are balanced across the layers; however, sparse per-layer interference or a dominant layer may increase the approximation error.

The accuracy can also be interpreted using the equivalent Gamma shape parameter $k_{\Gamma }=\frac{{\bar{I}}_{B}^{2}}{\sigma_{I,B}^{2}}$. The skewness and excess kurtosis of the moment-matched Gamma distribution are $2/\sqrt{k_{\Gamma }}$ and $6/k_{\Gamma }$, respectively. Therefore, as $k_{\Gamma }$ increases, both quantities approach zero and the Gamma distribution approaches a Gaussian distribution. Accordingly, the Gaussian approximation is mainly applicable to dense multi-layer constellations with a sufficiently large $k_{\Gamma }$ and without a dominant interference contribution. In sparse or strongly heterogeneous scenarios, the Gamma distribution should be retained to obtain more accurate tail probabilities. Therefore, under the above applicability conditions, the moment-matched Gamma distribution of the aggregate interference at the base station can be further approximated by the Gaussian distribution $N({\bar{I}}_{B},\sigma_{I,B}^{2})$. Since the aggregate interference received by the warden has the same mathematical form, the above approximation analysis also applies to $I_{W}$, which can be approximated by $N({\bar{I}}_{W},\sigma_{I,W}^{2})$.

Hence, the covert communication capacity of the satellite-to-ground link is defined as the maximum transmission rate $V_{LB}$ from satellite $L_{T}$ to base station *B* under dual constraints of detection probability $P_{det}$ (with threshold ϵ) and outage probability $P_{out}$ (with threshold κ):

(12) $$C_{L}=\max_{P_{L}}V_{LB}$$

(13) $$s.t.\;P_{\det}\left(P_{L}\right)\leq \epsilon \;\;\mathrm{and}\;\;P_{\mathrm{out}}\left(P_{L},V_{LB}\right)\leq \kappa$$

where the detection probability and outage probability formulas are

(14) $$P_{det}\left(P_{L}\right)=P\left({\bar{I}}_{W}-\frac{G_{L}G_{W}P_{L}{\left|h_{LW}\right|}^{2}r_{LW}^{-\alpha }}{2}<I_{W}<{\bar{I}}_{W}+\frac{G_{L}G_{W}P_{L}{\left|h_{LW}\right|}^{2}r_{LW}^{-\alpha }}{2}\right),$$

(15) $$P_{out}\left(P_{L},V_{LB}\right)=P\left(\log\left(1+\frac{G_{L}G_{B}P_{L}{\left|h_{LB}\right|}^{2}r_{LB}^{-\alpha }}{\sigma_{B}^{2}+I_{B}}\right)<V_{LB}\right).$$

Under the Gaussian approximation, the aggregate interference received by the satellite warden follows

$$I_{W}∼N\left({\bar{I}}_{W},\sigma_{I,W}^{2}\right).$$

The difference between the mean received powers under the transmission and non-transmission hypotheses is

$$\Delta_{W}=G_{L}G_{W}P_{L}r_{LW}^{-2}.$$

Accordingly, the detection probability of the satellite warden can be expressed as

$$P_{\det}\left(P_{L}\right)=2\Phi \left(\frac{\Delta_{W}}{2\sigma_{I,W}}\right)-1,$$

The detection constraint $P_{\det}\left(P_{L}\right)\leq \epsilon$ therefore yields

$$P_{L}\leq \frac{2\sigma_{I,W}\Phi^{-1}\left(\frac{1+\epsilon }{2}\right)}{G_{L}G_{W}r_{LW}^{-2}}.$$

Meanwhile, by approximating the aggregate interference at the base station using its mean ${\bar{I}}_{B}$, the outage probability of the satellite-to-base-station link is given by

$$P_{\mathrm{out}}=1-\exp\left[-\frac{\left(e^{V_{LB}}-1\right)\left(\sigma_{B}^{2}+{\bar{I}}_{B}\right)}{G_{L}G_{B}P_{L}r_{LB}^{-2}}\right].$$

The outage constraint $P_{\mathrm{out}}\leq \kappa$ leads to

$$V_{LB}\leq \ln\left[1+\frac{-G_{L}G_{B}P_{L}r_{LB}^{-2}\ln\left(1-\kappa \right)}{\sigma_{B}^{2}+{\bar{I}}_{B}}\right].$$

By substituting the maximum allowable transmit power imposed by the detection constraint into the above expression, the covert capacity can be approximated as

$$C_{L}\approx \ln\left[1+\frac{2G_{B}\sigma_{I,W}r_{LB}^{-2}}{G_{W}r_{LW}^{-2}\left(\sigma_{B}^{2}+{\bar{I}}_{B}\right)}\times \Phi^{-1}\left(\frac{1+\epsilon }{2}\right)\left[-\ln(1-\kappa )\right]\right].$$

For the interference-dominated scenario with ${\bar{I}}_{B}\gg \sigma_{B}^{2}$, and for sufficiently small ϵ and κ, we have

$$\Phi^{-1}\left(\frac{1+\epsilon }{2}\right)\approx \sqrt{\frac{\pi }{2}}\epsilon ,\;-\ln\left(1-\kappa \right)\approx \kappa ,$$

and ln(1+x)≈x. Therefore, the covert capacity can be further simplified as

(16) $$C_{L}\approx \frac{\sqrt{2\pi }\;G_{B}\sigma_{I,W}r_{LB}^{-2}}{G_{W}{\bar{I}}_{B}r_{LW}^{-2}}\epsilon \kappa .$$

### 3.3. Ground Routing Covert Performance

For the ground self-organizing routing stage, let $X_{S_{k}}$ and $X_{J_{i}}$ denote the signals to be transmitted by node $S_{k}$ and jammer $J_{i}$, respectively. The wireless channel is characterized by large-scale path loss with path-loss exponent α. Moreover, both the legitimate transmission signal and the jamming signal are modeled as Gaussian random variables, i.e., $X_{S_{k}}∼N\left(0,1\right)$ and $X_{J_{i}}∼N\left(0,\delta_{J_{i}}^{2}\right)$. Let $P_{J_{i}}$ denote the jamming power of $J_{i}$ and $Y_{W_{i}}$ denote the received signal at warden $W_{i}$. When no legitimate transmission is performed, the received signal at $W_{i}$ is given by

(17) $$Y_{W_{i}}=\sum_{i=1}^{M}\frac{\sqrt{P_{J_{i}}}}{d_{J_{i},W_{i}}^{\alpha /2}}\;X_{J_{i}}.$$

By contrast, when a legitimate transmission occurs, the received signal at $W_{i}$ becomes

(18) $$Y_{W_{i}}=\sum_{i=1}^{M}\frac{\sqrt{P_{J_{i}}}}{d_{J_{i},W_{i}}^{\alpha /2}}X_{J_{i}}+\frac{\sqrt{P}}{d_{S_{k},W_{i}}^{\alpha /2}}X_{S_{k}}.$$

We consider a candidate route $\Gamma =\{l_{1},l_{2},\ldots ,l_{K}\}$ connecting *S* and *D* under a general jamming configuration, where the jamming power $P_{J_{i}}$ of each jammer $J_{i}$ can be flexibly determined. The objective is to evaluate the end-to-end covertness of message delivery along this route. During the transmission process, wardens observe the signals emitted over the route and infer whether a legitimate transmission is taking place. Following the standard signal detection framework, we assume that the wardens employ the optimal hypothesis testing rule, which has been widely adopted in detection and estimation problems [28]. Let $P_{0}$ and $P_{1}$ denote the joint probability distribution functions of wardens’ observations when there is no transmission and when the transmission happens, respectively. According to Pinsker’s inequality [36], we have

(19) $$P_{FA}+P_{MD}\geq 1-\sqrt{\frac{1}{2}D_{e}\left(P_{1}∥P_{0}\right)},$$

where $P_{FA}$ denotes the false alarm probability, i.e., the probability that a warden declares the presence of a transmission when no transmission occurs, and $P_{MD}$ denotes the missed detection probability, i.e., the probability that a warden fails to detect an ongoing transmission. It follows from (19) that reducing the relative entropy $D_{e}$ increases the lower bound on the wardens’ total detection error probability, thereby improving the covertness of the transmission.

Based on the above transmission model, $P_{0}$ and $P_{1}$ follow multivariate Gaussian distributions with covariance matrices $\Sigma_{0}$ and $\Sigma_{1}$, and mean vectors $\mu_{0}$ and $\mu_{1}$, respectively. Using the result in [28], the relative entropy $D_{e}$ can be written as

(20) $$D_{e}\left(P_{1}∥P_{0}\right)=\frac{1}{2}\left[\mathrm{Tr}\left(\Sigma_{0}^{-1}\Sigma_{1}\right)+{\left(\mu_{0}-\mu_{1}\right)}^{T}\Sigma_{0}^{-1}\left(\mu_{0}-\mu_{1}\right)-\dim\left(\Sigma_{0}\right)-\ln\frac{\det(\Sigma_{1})}{\det(\Sigma_{0})}\right].$$

Since the wardens jointly observe the transmission behavior over *K* links and there are *M* wardens in total, each observation vector contains K×M components. Consequently, both $\Sigma_{0}$ and $\Sigma_{1}$ are KM×KM covariance matrices.

In the absence of legitimate transmission, the wardens receive only jamming signals. Therefore, from Equation (17), the covariance matrix under the null hypothesis is obtained as

(21) $$\Sigma_{0}=\mathrm{diag}\left(\underset{K\;\mathrm{times}}{\underset{︸}{J_{W_{1}},\ldots ,J_{W_{1}}}},\ldots ,J_{W_{M}}\right),$$

where $J_{W_{i}}$ represents the jamming power observed at $W_{i}$. Since the interference received by a warden is dominated by its associated nearby jammer, while the impact of other jammers is relatively weak, $J_{W_{i}}$ can be approximated by

(22) $$J_{W_{i}}\approx \frac{P_{J_{i}}}{d_{J_{i},W_{i}}^{\alpha }}\;\delta_{J_{i}}^{2}.$$

When legitimate transmission is present, the covariance matrix under the alternative hypothesis can be expressed as

(23) $$\Sigma_{1}=\Sigma_{0}+uu^{T},$$

where u is a column vector given by

(24) $$u={\left[\frac{\sqrt{P}}{d_{S_{1},W_{1}}^{\alpha /2}},\frac{\sqrt{P}}{d_{S_{2},W_{1}}^{\alpha /2}},\ldots ,\frac{\sqrt{P}}{d_{S_{K},W_{1}}^{\alpha /2}},\ldots ,\frac{\sqrt{P}}{d_{S_{1},W_{2}}^{\alpha /2}},\ldots ,\frac{\sqrt{P}}{d_{S_{1},W_{M}}^{\alpha /2}},\ldots ,\frac{\sqrt{P}}{d_{S_{K},W_{M}}^{\alpha /2}}\right]}^{T}.$$

Since $\mu_{0}$ and $\mu_{1}$ are zero vectors, substituting (21) and (23) into (20), followed by standard linear algebra manipulations, yields

(25) $$D_{e}\left(P_{1}∥P_{0}\right)=\frac{1}{2}\left[\sum_{i=1}^{M}\sum_{k=1}^{K}\frac{P}{J_{W_{i}}d_{S_{k},W_{i}}^{\alpha }}-\ln\left(1+\sum_{i=1}^{M}\sum_{k=1}^{K}\frac{P}{J_{W_{i}}d_{S_{k},W_{i}}^{\alpha }}\right)\right].$$

## 4. Incentivized Ground Routing Design

Although the physical information flow proceeds from the LEO satellite to a selected ground base station and then to the final destination, the corresponding design problem can be more conveniently solved in a backward order. Specifically, the utility of a candidate base station is determined not only by the satellite-to-ground covert capacity, but also by the best ground route that can be established from this base station to the destination. Therefore, we adopt a decomposition-based backward design principle: for a given satellite-to-base station pairing, we first investigate the ground routing problem from the selected base station to the destination. The optimized ground-routing utility obtained in this stage will then be used as the ground-side payoff when performing satellite-to-base station selection.

Given a fixed satellite-to-ground base station association, the base station serves as the ground source and forwards the message to the destination through multi-hop self-organizing routing. In this stage, the covertness of each candidate route is strongly affected by the artificial jamming power provided near the wardens. However, the jammers are self-interested entities and consume their own energy to generate jamming signals. Therefore, the routing decision cannot be separated from the incentive provided to the jammers. A route that appears favorable without considering jammer participation may become inefficient if it requires excessive reward expenditure to induce sufficient jamming power. Motivated by this observation, we develop an incentive-aware route design method in which the source jointly accounts for route-dependent covertness enhancement and the reward cost required to stimulate cooperative jamming.

We first analyze the incentive interaction on a fixed ground route. The source allocates a total reward to the recruited jammers, while each jammer determines its jamming power according to the reward competition and its own power cost. This interaction naturally forms a leader-follower structure: the source first determines the total reward, and the jammers subsequently choose their jamming powers in response. The resulting outcome is then used as the basis for constructing a route weight, so that route selection can be converted into a shortest-weighted path search.

For a given route, the source prefers a smaller relative entropy, since a lower value of the covertness metric makes the wardens’ observations less distinguishable and hence improves the covert performance. To capture this preference while considering the payment to jammers, the source utility is defined as

(26) $$U_{S}=\lambda e^{-2D_{e}}-R,$$

where λ is a system parameter. The first term represents the benefit obtained from improving covertness, while the second term accounts for the total reward paid by the source. This utility decreases with the covertness metric $D_{e}$, and the exponential form emphasizes the utility loss caused by the degradation of covertness when the route is already relatively covert.

The reward received by each jammer is determined according to its relative contribution to the artificial jamming observed by the wardens. Since jammer $J_{i}$ is deployed to mainly interfere with the corresponding warden $W_{i}$, its contribution is evaluated by the product of its jamming power and the corresponding channel-dependent contribution factor $r_{i}=d_{J_{i},W_{i}}^{-\alpha }$. The cost incurred by jammer $J_{i}$ is proportional to its jamming power, namely $c\cdot P_{J_{i}}$, where c>0 denotes the unit cost of jamming power. Therefore, the utility of jammer $J_{i}$ is given by (We define the reward share of each jammer as zero when the total effective jamming contribution is zero. Under this definition, the all-zero power profile yields zero utility for every jammer. When R>0 and M≥2, however, the all-zero profile cannot be a Nash equilibrium, since any jammer can obtain a positive utility by unilaterally selecting a sufficiently small positive power.)

(27) $$U_{J_{i}}=\frac{P_{J_{i}}r_{i}}{\sum_{J_{j}\in J}P_{J_{j}}r_{j}}R-cP_{J_{i}}.$$

For a fixed reward *R*, the jammers compete for the reward by independently selecting their jamming powers (Although the jamming-power strategy space is unbounded, no explicit maximum-power constraint is required. The proportional reward of each jammer is upper-bounded by *R*, whereas its power cost increases linearly with $P_{J_{i}}$. Therefore, $\lim_{P_{J_{i}}\to \infty }U_{J_{i}}=-\infty$, implying that an arbitrarily large jamming power can never maximize the jammer utility. For any positive aggregate contribution from the other jammers, the utility is strictly concave in the jammer’s own power and admits a unique finite maximizer.) This jammer-side interaction is modeled as a non-cooperative game. A stable power configuration is reached when no jammer can improve its own utility by unilaterally changing its jamming power. Specifically, a strategy profile $P_{J}^{ne}=\left(P_{J_{1}}^{ne},P_{J_{2}}^{ne},\ldots ,P_{J_{M}}^{ne}\right)$ is a Nash equilibrium if, for any jammer $J_{i}$ and any feasible strategy $P_{J_{i}}\geq 0$, the following condition holds:

(28) $$U_{J_{i}}\left(P_{J_{i}}^{ne},P_{-J_{i}}^{ne}\right)\geq U_{J_{i}}\left(P_{J_{i}},P_{-J_{i}}^{ne}\right).$$

For M≥2, to establish the existence and uniqueness of the Nash equilibrium, define $x_{i}=r_{i}P_{J_{i}}$ as the effective jamming contribution of jammer $J_{i}$, and let $a_{i}=c/r_{i}$ denote its effective unit cost. The utility of jammer $J_{i}$ can then be rewritten as

$$U_{J_{i}}\left(x_{i},x_{-i}\right)=\frac{Rx_{i}}{x_{i}+X_{-i}}-a_{i}x_{i},$$

where $X_{-i}=\sum_{j\neq i}x_{j}$. For any $X_{-i}>0$, the first- and second-order derivatives with respect to $x_{i}$ are

$$\frac{\partial U_{J_{i}}}{\partial x_{i}}=\frac{RX_{-i}}{{\left(x_{i}+X_{-i}\right)}^{2}}-a_{i},$$

$$\frac{\partial^{2}U_{J_{i}}}{\partial x_{i}^{2}}=-\frac{2RX_{-i}}{{\left(x_{i}+X_{-i}\right)}^{3}}<0.$$

Therefore, the utility of each jammer is strictly concave in its own strategy, and each jammer has a unique best response for any fixed strategies of the other jammers. Moreover, an equilibrium cannot contain only one active jammer, because such a jammer could continuously reduce its jamming power while still receiving the entire reward.

Let $J_{a}$ denote the set of active jammers, $m=|J_{a}|\geq 2$, and $X=\sum_{j\in J_{a}}x_{j}$. For each active jammer, the first-order equilibrium condition is

$$\frac{R\left(X-x_{i}\right)}{X^{2}}=a_{i}.$$

Summing the above condition over all active jammers yields

$$X^{\mathrm{ne}}=\frac{R(m-1)}{\sum_{j\in J_{a}}a_{j}}.$$

Consequently, the equilibrium effective contribution of jammer $J_{i}$ is

(29) $$x_{i}^{\mathrm{ne}}=X^{\mathrm{ne}}\left(1-\frac{\left(m-1\right)a_{i}}{\sum_{j\in J_{a}}a_{j}}\right),\;i\in J_{a}.$$

A jammer is active if $a_{i}<\frac{\sum_{j\in J_{a}}a_{j}}{m-1}$, whereas an inactive jammer satisfies the reverse weak inequality. By arranging the effective costs in ascending order and retaining the largest set satisfying the above participation condition, the active-jammer set is uniquely determined. Since the active set is unique and every active jammer has a unique best response, the resulting Nash equilibrium is unique.

When all recruited jammers satisfy the participation condition, i.e., $r_{i}\sum_{j=1}^{M}\frac{1}{r_{j}}>M-1$, all *M* jammers are active. Substituting $a_{i}=c/r_{i}$ and $x_{i}=r_{i}P_{J_{i}}$ into the above equilibrium expression gives

(30) $$P_{J_{i}}^{\mathrm{ne}}=R\cdot \kappa_{i},$$

where $\kappa_{i}$ is expressed as

(31) $$\kappa_{i}={\left[\frac{\left(M-1\right)\left(\sum_{j=1}^{M}\frac{r_{i}}{r_{j}}-M+1\right)}{c\cdot {\left(\sum_{j=1}^{M}\frac{r_{i}}{r_{j}}\right)}^{2}}\right]}^{+}.$$

This result shows that, once the reward *R* is fixed, the jamming response of each jammer can be directly obtained from its contribution factor and cost parameter.

**Remark** **1.** *When M=1, the proportional reward competition degenerates because the single jammer receives the entire reward for any strictly positive power. Consequently, no pure-strategy equilibrium exists for R>0 under the utility in *(27)*. Therefore, the proposed competition mechanism is applied to scenarios with M≥2. A single-jammer scenario can instead be handled through direct power procurement and bilateral compensation.*

We next determine the reward selected by the source. By substituting the equilibrium jamming power response into the source utility, the route-dependent term can be summarized as

(32) $$\Delta =\sum_{i=1}^{M}\sum_{k=1}^{K}\frac{Pd_{J_{i},W_{i}}^{\alpha }}{\kappa_{i}\delta_{J_{i}}^{2}d_{S_{k},W_{i}}^{\alpha }}.$$

Then, the source utility becomes a single-variable function of the reward

(33) $$U_{S}\left(R\right)=\lambda \frac{1+\frac{\Delta }{R}}{e^{\frac{\Delta }{R}}}-R.$$

Taking the first- and second-order derivatives of $U_{S}$ w.r.t. *R*, we have

(34) $$\begin{array}{cc} \frac{\partial U_{S}}{\partial R} & =\frac{\lambda \Delta^{2}}{R^{3}e^{\frac{\Delta }{R}}}-1, \end{array}$$

(35) $$\begin{array}{cc} \frac{\partial^{2}U_{S}}{\partial R^{2}} & =-\frac{\lambda \Delta^{2}}{{\left(R^{3}e^{\frac{\Delta }{R}}\right)}^{2}}R\left(3R-\Delta \right)e^{\frac{\Delta }{R}}. \end{array}$$

To determine the globally optimal reward, the boundary solution should also be considered. Although the utility function in (33) is defined for R>0, it admits the following continuous extension at R=0

(36) $$U_{S}\left(0\right)=\lim_{R\to 0^{+}}U_{S}\left(R\right)=0.$$

Let $g\left(R\right)≜\frac{\partial U_{S}}{\partial R}$, according to (34), g(R) approaches −1 as $R\to 0^{+}$ and as R→∞. Moreover, g(R) reaches its unique maximum at R=Δ/3, and the corresponding maximum value is

(37) $$g\left(\frac{\Delta }{3}\right)=\frac{27\lambda }{e^{3}\Delta }-1.$$

Therefore, the optimal reward can be determined according to the following two cases. If $\frac{27\lambda }{e^{3}\Delta }\leq 1$, then g(R)≤0 for all R>0. In this case, the source utility is non-increasing over R≥0, and the globally optimal reward is the boundary solution $R^{*}=0$. If $\frac{27\lambda }{e^{3}\Delta }>1$, then (34) has two positive roots, denoted by $R_{1}$ and $R_{2}$, which satisfy $R_{1}<\frac{\Delta }{3}<R_{2}$.

The derivative changes from negative to positive at $R_{1}$ and from positive to negative at $R_{2}$. Hence, $R_{1}$ corresponds to a local minimum, whereas $R_{2}$ corresponds to a local maximum. Nevertheless, the local maximum should still be compared with the boundary solution R=0. Consequently, the globally optimal reward is given by

(38) $$R^{*}=\left\{\begin{array}{cc} R_{2}, & U_{S}\left(R_{2}\right)>U_{S}\left(0\right),\\ 0, & U_{S}\left(R_{2}\right)\leq U_{S}\left(0\right). \end{array}\right.$$

Since $U_{S}\left(0\right)=0$, a positive reward is allocated only when the larger stationary point produces a strictly positive source utility.

In numerical implementation, the larger root $R_{2}$ is computed only when $27\lambda /(e^{3}\Delta )>1$. Since $R_{2}>\Delta /3$, a one-dimensional bracketing method, such as the bisection method or Brent’s method, can be applied over the interval $\left(\Delta /3,R_{\mathrm{up}}\right)$. The upper bound $R_{\mathrm{up}}$ is progressively enlarged until $g(R_{\mathrm{up}})<0$. If no positive stationary point exists, or if the utility evaluated at $R_{2}$ is not greater than zero, the reward is directly set to $R^{*}=0$.

When $R^{*}>0$, the corresponding maximum source utility is

(39) $$U_{S}^{*}=\lambda \frac{1+\frac{\Delta }{R^{*}}}{e^{\frac{\Delta }{R^{*}}}}-R^{*},$$

and the resulting optimal jamming power of jammer $J_{i}$ is obtained as $P_{J_{i}}^{*}=R^{*}\cdot \kappa_{i}$. Thus, the reward decision of the source and the jamming-power decisions of the jammers are jointly determined for the considered route.

The above analysis also provides a direct criterion for route selection. For different candidate routes, the optimized source utility is determined by the route-dependent value Δ. Since a smaller Δ leads to a larger optimized source utility, selecting the best ground route is equivalent to finding the route with the minimum accumulated route cost. Let L denote the set of available routes between the source and the destination. The optimal route is then obtained as

(40) $$\begin{array}{cc} \Pi^{opt} & =\arg\max_{\Pi \in L}U_{S}\left(\Pi \right)=\arg\min_{\Pi \in L}\Delta \left(\Pi \right)\\ \; & =\arg\min_{\Pi \in L}\sum_{i=1}^{M}\sum_{k=1}^{K}\frac{Pd_{J_{i},W_{i}}^{\alpha }}{\kappa_{i}\delta_{J_{i}}^{2}d_{S_{k},W_{i}}^{\alpha }}=\arg\min_{\Pi \in L}\sum_{k=1}^{K}\Theta_{k}, \end{array}$$

where $\Theta_{k}$ is given by

(41) $$\Theta_{k}=\sum_{i=1}^{M}\frac{Pd_{J_{i},W_{i}}^{\alpha }}{\kappa_{i}\delta_{J_{i}}^{2}d_{S_{k},W_{i}}^{\alpha }}.$$

Equation (40) indicates that the incentive-aware route selection problem can be interpreted as a shortest-weighted path problem. Each candidate link is assigned a weight according to Equation (41), which reflects the reward-induced jamming cost required to maintain covertness over that link. A link with a smaller weight is more favorable because it can achieve the desired covertness with a lower incentive burden. Therefore, the source can apply a classical shortest-path algorithm, such as Dijkstra’s algorithm or the Bellman-Ford algorithm [37], to identify the route with the minimum accumulated weight. After the route is selected, the source determines the optimal reward and the corresponding jamming powers. The overall procedure is summarized in Algorithm 1.
**Algorithm 1** Incentivized Ground Route Selection for Covert Multi-hop Forwarding  1:**Input:** Network topology and basic system parameters $\left\{P,\alpha ,c,\lambda ,\delta_{J_{i}}\right\}$;  2:**Output:** Optimal path $\Pi^{opt}$, optimal configuration of source rewards $R^{*}$ and jamming power $P_{J}^{*}$;  3:Evaluate the weight Θ of every feasible link *l* using Equation (41);  4:Run a shortest-path search, e.g., Dijkstra’s algorithm, from the source to the destination and obtain $\Pi^{opt}$;  5:For $\Pi^{opt}$, compute the larger zero of (34) and assign it to $R^{*}$;  6:Compute $\zeta =27\lambda /(e^{3}\Delta )-1$;  7:**if** ζ≤0 **then**  8:    Set $R^{*}=0$;  9:**else**10:    Numerically compute the larger root $R_{2}>\Delta /3$ of (34) using a bracketing root-finding method;11:    **if** $U_{S}\left(R_{2}\right)>0$ **then**12:        Set $R^{*}=R_{2}$;13:    **else**14:        Set $R^{*}=0$;15:    **end if**16:**end if**17:**if** $R^{*}=0$ **then**18:    Set $P_{J_{i}}^{\mathrm{ne}}=0$, ∀i∈J;19:**else**20:    Each jammer sets $P_{J_{i}}^{*}$ according to Equation (30);21:**end if**22:**return** $\left\{\Pi^{opt},R^{*},P_{J}^{*}\right\}$;

For Algorithm 1, let $N_{g}$ and $E_{g}$ denote the numbers of legitimate nodes and feasible links in the ground network, respectively. Since the weight of each feasible link is calculated by summing the contributions of *M* wardens/jammers, the link-weight calculation requires $O(ME_{g})$ operations. The resulting shortest-weighted path can be obtained using Dijkstra’s algorithm with a binary heap in $O(\left(N_{g}+E_{g}\right)\log N_{g})$ time. The optimal reward $R^{*}$ is determined by solving the scalar equation in (34), which can be efficiently implemented using a one-dimensional numerical search. The equilibrium jamming powers are then directly obtained from (30) in O(M) time. Therefore, the dominant computational complexity of Algorithm 1 is $O(ME_{g}+\left(N_{g}+E_{g}\right)\log N_{g})$. Because the jammer responses are available in closed form and the routing stage uses a finite shortest-path procedure, Algorithm 1 terminates in a finite number of steps and does not require an iterative game-convergence process.

## 5. Zero-Determinant Strategy-Based Base Station Selection

In the satellite-to-ground communication link, base station selection plays a critical role in determining the long-term covert communication performance. In the system model presented in Section 3, the covert capacity of the satellite-to-ground link is evaluated under the conservative assumption that the satellite warden is located near the selected base station and thus falls within the high-gain reception region of the LEO satellite’s directional beam. This assumption is useful for characterizing the instantaneous worst-case covert performance. However, in long-term covert communication, the satellite transmitter does not necessarily have to keep using the same base station. Instead, it can dynamically switch among candidate base stations over different communication cycles, thereby reducing the opportunity for the warden to continuously approach and monitor the actual relay base station.

**Figure 3:**
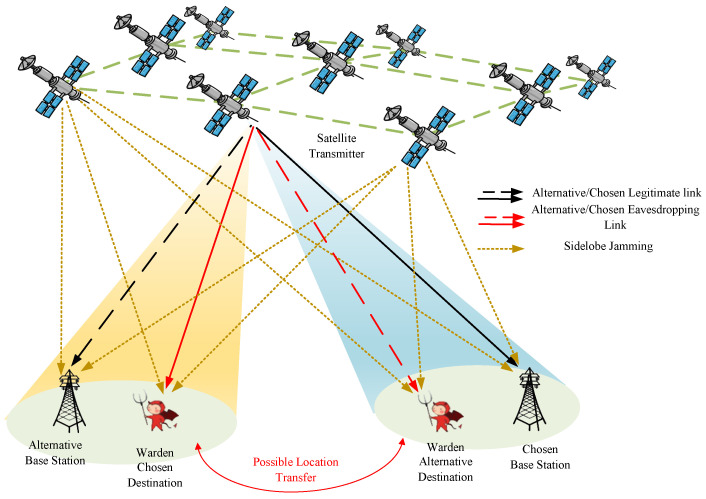
One round of the repeated base-station selection game between the LEO satellite and the satellite warden.

Motivated by this observation, we model the long-term interaction between the LEO satellite transmitter and the satellite warden as a repeated base-station selection game. In each communication cycle, the satellite transmitter selects one ground base station as the relay, while the warden chooses one base station to monitor or approach. The resulting payoff depends on whether the warden’s monitoring decision matches the satellite’s relay selection, as well as on the corresponding satellite-to-ground covert capacity and ground-routing utility. As illustrated in Figure 3, the satellite transmitter and the warden make their decisions repeatedly over time, and the outcome of each round affects the state evolution of the game.

Since there are $N_{b}$ available base stations in the system, the total number of possible outcome combinations in each round of the game is $\Lambda =N_{b}^{2}$. These outcomes can be represented as

(42) $$e_{L}e_{W}=\left\{\underset{g_{1}}{\underset{︸}{11}},\;\ldots ,\underset{g_{N_{b}}}{\underset{︸}{1N_{b}}},\;\ldots ,\underset{g_{\zeta }}{\underset{︸}{N_{b}N_{b}}}\right\},$$

where $e_{L}$ and $e_{W}$ represent the base station selections of the satellite transmitter and the warden, respectively. The base station numbers selected by both parties form $g_{k}$, representing a game outcome where the satellite transmitter selects base station $B_{i}$ as the relay for forwarding, while the warden chooses to approach $B_{j}$ for eavesdropping.

**Figure 4:**
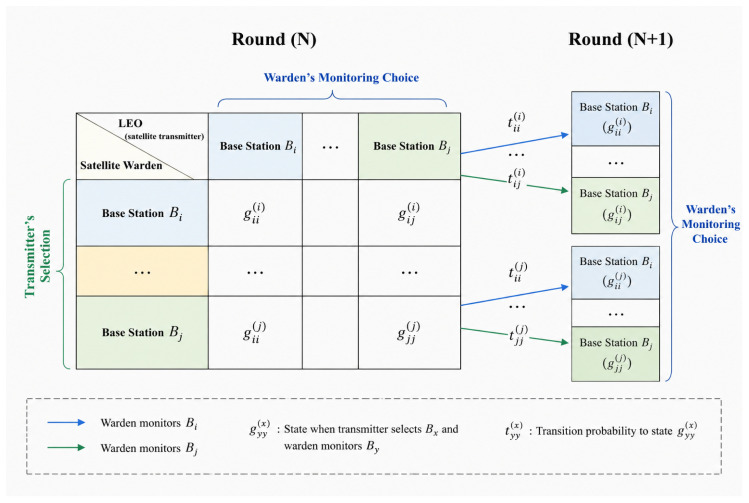
State transition illustration of the repeated base-station selection game.

To capture the dependence of the current decision on the previous interaction outcome, we adopt a memory-one strategy for the satellite transmitter. Specifically, the memory-one ZD strategy of the satellite transmitter is denoted by $s=(s_{1},s_{2},\ldots ,s_{\Lambda })$, where $s_{k}$ denotes the probability that the satellite transmitter selects the target relay base station in the current round conditioned on the *k*-th outcome in the previous round. The warden’s monitoring rule is denoted by $b=(b_{1},b_{2},\ldots ,b_{N_{b}})$, where $b_{j}$ is the probability that the warden monitors base station $B_{j}$ in the current round. This formulation is general enough to include both random monitoring and fixed monitoring of a specific base station as special cases. Figure 4 illustrates the state transition process within one round of the repeated game.

Let $Q={\left[q_{ij}\right]}_{\Lambda \times \Lambda }$ denote the one-step state transition matrix of the game, which is given by

(43) $$q_{k,(i,j)}=a_{k,i}b_{j},\;k=1,\ldots ,\Lambda ,$$

where (i,j) denotes the next-round outcome in which the satellite transmitter selects $B_{i}$ and the warden monitors $B_{j}$. If $B_{r}$ is the target relay base station, then $a_{k,r}=s_{k}$ and $a_{k,i}=\left(1-s_{k}\right)/\left(N_{b}-1\right)$ for i≠r. Each row of Q therefore sums to one.

**Assumption** **1** (Ergodic repeated interaction). *For each considered target base station and each admissible strategy pair (s,b), the transition matrix Q(s,b) induces an irreducible and aperiodic finite-state Markov chain.*

This assumption ensures that the repeated base-station selection game admits a unique stationary distribution. A sufficient condition is that the effective strategies of both players have full support, such that every feasible action is selected with a strictly positive probability. Under this condition, every outcome can be reached from any current outcome with positive probability, making Q a positive stochastic matrix and, consequently, irreducible and aperiodic.

The long-term behavior of the repeated game is characterized by the stationary distribution of the induced Markov chain. Let v denote the stationary vector of Q, and define matrix $Q^{\prime }=Q-I$ with I being the identity matrix of the same size as Q. Then, we have

(44) $$v^{T}\cdot Q=v^{T},\;v^{T}Q^{\prime }=0.$$

Applying Cramer’s rule [38] to $Q^{\prime }$ yields

(45) $$\mathrm{Adj}\left(Q^{\prime }\right)Q^{\prime }=\det\left(Q^{\prime }\right)I=0,$$

where $\mathrm{Adj}(Q^{\prime })$ denotes the adjugate matrix of $Q^{\prime }$. Equations (44) and (45) imply that v is proportional to each row of $\mathrm{Adj}(Q^{\prime })$. Let $Q^{\prime }=\left[q_{1},q_{2},\ldots ,q_{\Lambda }\right]$, where $q_{i}={\left[q_{1i}^{\prime },q_{2i}^{\prime },\ldots ,q_{\Lambda i}^{\prime }\right]}^{T}$ is a vector consisting of the *i*-th column of $Q^{\prime }$. Thus, for the dot product of any vector $f={\left[f_{1},f_{2},\ldots ,f_{\Lambda }\right]}^{T}$ with the stable vector v, there is

(46) $$v\cdot f=\det\left[q_{1},q_{2},\ldots ,q_{\Lambda -1},f\right].$$

The key idea of the ZD strategy is to use the satellite-controlled memory-one probabilities to enforce a linear relation between the long-term expected payoffs of the satellite transmitter and the warden. To construct such a relation, the entries controlled by the satellite transmitter are collected into an affine column through elementary column operations on $Q^{\prime }$. For the target base-station outcome set $K_{r}$, this controllable column can be written as

(47) $$q_{ZD}^{new}={\left[s_{1}-I_{1\in K_{r}},\ldots ,s_{\Lambda }-I_{\Lambda \in K_{r}}\right]}^{T}.$$

Since elementary transformations of a matrix cannot alter its determinant value, we can get

(48) $$v\cdot f=\det\left[q_{1},\ldots ,q_{ZD}^{new},\ldots ,q_{\Lambda -1},f\right].$$

We define the payoff vectors of the satellite transmitter and the warden as $U_{T}=\left[U_{T}^{1},U_{T}^{2},\ldots ,U_{T}^{\Lambda }\right]$ and $U_{W}=\left[U_{W}^{1},U_{W}^{2},\ldots ,U_{W}^{\Lambda }\right]$, respectively. Here, $U_{T}^{k}$ and $U_{W}^{k}$ are the payoffs of the two players under the *k*-th outcome in the base-station selection game. Then, for X∈{T,W}, we have

(49) $${\bar{U}}_{X}=\frac{v\cdot U_{X}}{v\cdot 1}=\frac{\det\left[q_{1},\ldots ,q_{\Lambda -1},U_{X}\right]}{\det\left[q_{1},\ldots ,q_{\Lambda -1},1\right]},$$

where 1 is the vector with all elements being 1.

To clarify how the ZD strategy imposes a linear relation between the long-term expected payoffs, we define $D\left(f\right)=\det\left[q_{1},\ldots ,q_{ZD}^{new},\ldots ,q_{\Lambda -1},f\right]$, where f is an arbitrary column vector. According to Equation (49), the expected payoff of player X∈{T,W} is given by

(50) $${\bar{U}}_{X}=\frac{D(U_{X})}{D(1)}.$$

Therefore, the weighted combination of the transmitter and warden payoffs can be expressed as

(51) $${\bar{U}}_{T}+\chi {\bar{U}}_{W}-\beta =\frac{D\left(U_{T}\right)+\chi D\left(U_{W}\right)-\beta D\left(1\right)}{D(1)}=\frac{D(U_{T}+\chi U_{W}-\beta 1)}{D(1)}.$$

As shown in Equation (47), the transmitter can control the column $q_{ZD}^{new}$ through its memory-one strategy s. The transmitter therefore selects s such that

(52) $$q_{ZD}^{new}=\phi \left(U_{T}+\chi U_{W}-\beta 1\right),$$

where ϕ≠0 is a scaling factor chosen to ensure that all strategy probabilities lie within [0,1]. Under this construction, the last column in the numerator of Equation (50) is proportional to the controllable ZD column. The determinant thus contains two linearly dependent columns and satisfies

$$D(U_{T}+\chi U_{W}-\beta 1)=0.$$

Consequently, we have

(53) $${\bar{U}}_{T}+\chi {\bar{U}}_{W}-\beta =0.$$

Hence, the transmitter can enforce the following weighted long-term payoff relation independently of the warden’s monitoring rule

(54) $$U_{all}={\bar{U}}_{T}+\chi {\bar{U}}_{W}=\beta .$$

The above relation indicates that, once a feasible ZD strategy is constructed, the satellite transmitter can stabilize the weighted long-term utility independently of the warden’s monitoring rule. Therefore, the stable utility control problem can be formulated as

(55) $$\begin{array}{cc} \mathrm{SUC}:\;\max\; & \beta \end{array}$$

(56) $$\begin{array}{cc} s.t.\; & 0\leq s\leq 1, \end{array}$$

(57) $$\begin{array}{cc} \; & q_{ZD}^{new}=\phi \left(U_{T}+\chi U_{W}-\beta 1\right). \end{array}$$

However, such a ZD construction is not always feasible, since the memory-one vector must satisfy the probability constraint. For a target base station $B_{r}$, let $K_{r}$ denote the set of outcomes in which the satellite transmitter selects $B_{r}$, and let ${\bar{K}}_{r}$ denote the remaining outcomes. A feasible ZD probability vector exists only when the target outcomes are sufficiently separated from the non-target outcomes in the payoff matrix. To stabilize a high transmitter utility, a sufficient separation condition is

(58) $$\max_{k\in {\bar{K}}_{r}}U_{T}^{k}\leq \min_{k\in K_{r}}U_{T}^{k}.$$

To suppress the warden utility, the corresponding condition is

(59) $$\min_{k\in {\bar{K}}_{r}}U_{W}^{k}\geq \max_{k\in K_{r}}U_{W}^{k}.$$

Conditions (58) and (59) characterize the payoff separation between the target and non-target outcomes with respect to the transmitter and warden utilities, respectively. They provide sufficient conditions for the feasibility of the general weighted-payoff ZD construction.

It is worth noting that the feasibility conditions in (58) and (59) may not always hold. In particular, when the payoff ranges corresponding to the target and non-target base stations overlap, the desired ZD relation may lead to one or more memory-one probabilities outside the interval [0,1]. We therefore introduce a feasibility-aware backoff mechanism.

For a given target base station $B_{r}$, the transmitter first relaxes the desired payoff level and determines the largest feasible value of β by solving

(60) $$\begin{array}{cc} \max_{\beta ,\phi }\;\; & \beta \\ s.t.\;\; & 0\leq I_{k\in K_{r}}+\phi \left(U_{T}^{k}+\chi U_{W}^{k}-\beta \right)\leq 1,\;\forall k. \end{array}$$

If this problem is feasible, the resulting β and ϕ are used to construct the memory-one ZD strategy. This relaxation preserves the linear long-term payoff relation but may stabilize it at a lower target value than originally desired. If no feasible ZD strategy exists for the current target base station, the transmitter evaluates all candidate targets. Define the payoff-separation margins of base station $B_{r}$ as $\Delta_{T}\left(r\right)=\min_{k\in K_{r}}U_{T}^{k}-\max_{k\in {\bar{K}}_{r}}U_{T}^{k}$ and $\Delta_{W}\left(r\right)=\min_{k\in {\bar{K}}_{r}}U_{W}^{k}-\max_{k\in K_{r}}U_{W}^{k}$. A candidate target is ZD-feasible when $\Delta_{T}\left(r\right)\geq 0$ and $\Delta_{W}\left(r\right)\geq 0$. Among the feasible candidates, the transmitter selects the target with the largest robust payoff

(61) $$r^{*}=\underset{r:\;\Delta_{T}\left(r\right)\geq 0,\;\Delta_{W}\left(r\right)\geq 0}{\arg\max}\;\min_{j}U_{T}^{\left(r,j\right)}.$$

When no candidate base station satisfies the feasibility conditions, exact ZD payoff control is unavailable. In this case, the transmitter temporarily adopts a utility-aware randomized policy

(62) $$p_{i}=\frac{\exp\left(\tau {\underline{U}}_{T}\left(i\right)\right)}{\sum_{\ell =1}^{N_{b}}\exp\left(\tau {\underline{U}}_{T}\left(\ell \right)\right)},\;{\underline{U}}_{T}\left(i\right)=\min_{j}U_{T}^{\left(i,j\right)}.$$

Here, τ>0 controls the balance between utility preference and randomization. A larger τ places more probability on the base station with the largest robust payoff, whereas a smaller τ produces a more randomized selection policy. This fallback strategy does not enforce an exact linear payoff relation, but it avoids deterministic relay selection and limits the predictability of the transmitter’s behavior. The transmitter periodically rechecks the feasibility conditions and resumes the ZD strategy once a feasible target becomes available.

It should be emphasized that the construction in (52) and (60) represents the general ZD strategy, which controls the weighted long-term payoff relation ${\bar{U}}_{T}+\chi {\bar{U}}_{W}=\beta$. The closed-form strategy presented below focuses on transmitter-utility stabilization and is obtained by setting χ=0 and $\beta =\beta_{T}$. Therefore, it directly pins the transmitter’s long-term expected utility, whereas the warden’s utility is not independently controlled in this special case.

The resulting ZD-based base-station selection strategy is summarized in the following theorem.

**Theorem** **1.** *Under Assumption 1, by employing the ZD strategy summarized in Algorithm 2, the satellite transmitter can stabilize the long-term expected utility relation in the repeated base-station selection game. Specifically, for the target base station $B_{r}$, a feasible ZD strategy $s=(s_{1},s_{2},\ldots ,s_{\Lambda })$ can be expressed as*

(63) $$s_{k}=\left\{\begin{array}{cc} 1+\phi (U_{T}^{k}-\beta_{T}), & k\in K_{r},\\ \phi (U_{T}^{k}-\beta_{T}), & k\in {\bar{K}}_{r}, \end{array}\right.$$

*where $\beta_{T}$ is the target transmitter payoff and ϕ is selected such that $0\leq s_{k}\leq 1$ for all k. Under condition *(58)*, such a ϕ exists, while Assumption 1 guarantees that the induced Markov chain admits a unique stationary distribution. Consequently, the transmitter’s long-term expected utility converges to $\beta_{T}$, independently of the initial state and the admissible warden monitoring rule.*

For Algorithm 2, the repeated game contains $\Lambda =N_{b}^{2}$ states. Constructing the transition matrix $Q\in R^{\Lambda \times \Lambda }$ requires $O(\Lambda^{2})$ time and memory. When the stationary distribution is evaluated by power iteration, each iteration requires $O(\Lambda^{2})$ operations, and the total complexity after *I* iterations is $O\left(I\Lambda^{2}\right)=O\left(IN_{b}^{4}\right)$. The iteration can be terminated when $v^{\left(t+1\right)}-v^{\left(t\right)}\leq \varepsilon_{v}$, where $\varepsilon_{v}$ is a prescribed convergence tolerance. If the transition matrix induces an irreducible and aperiodic Markov chain, the stationary distribution is unique and the iteration converges from any initial distribution. Under this condition, the ZD construction enforces the linear long-term payoff relation derived in (53).
**Algorithm 2** ZD-Based Dynamic Base-Station Selection  1:**Input:** candidate base stations B, transmitter payoff vector $U_{T}$, warden payoff vector $U_{W}$, target base station $B_{r}$;  2:**Output:** feasible ZD strategy $s=(s_{1},s_{2},\ldots ,s_{\Lambda })$;  3:Construct the outcome set $\left\{\omega_{ij}\right\}$ and identify $K_{r}$ and ${\bar{K}}_{r}$;  4:Check the feasibility conditions in  (58) and (59) for the current target base station;  5:If feasible, solve the constrained ZD parameter problem and construct the ZD probability vector s;  6:Otherwise, examine the remaining candidate base stations and select a feasible target according to (61);  7:If no feasible target exists, apply the randomized backoff policy in (62);  8:Periodically re-evaluate the ZD feasibility conditions;  9:Construct the transition matrix Q under the warden monitoring rule b;10:Iterate the stationary distribution and compute ${\bar{U}}_{T}$ and ${\bar{U}}_{W}$ by (49);11:**return** s;

## 6. Simulation Results

This section evaluates the proposed two-stage satellite-ground covert routing framework by simulations. We first present the simulation setup and use a representative topology to illustrate the joint base-station selection and ground routing process. We then examine how satellite-to-ground and ground-network parameters affect the end-to-end utility, and compare the proposed joint selection rule with several baseline schemes. Finally, we evaluate the long-term base-station selection performance under the ZD strategy.

### 6.1. Simulation Setup and Joint Selection Example

The default simulation parameters are summarized in Table 1. All simulations were implemented in MATLAB R2026a. For the ground segment, the network is generated in a 50×50 square area. The destination is located at (25,25). Three candidate base stations are placed at (0,50), (25,0), and (50,50). The ground network contains 49 legitimate relay nodes and M=5 ground wardens. These nodes and wardens are independently and uniformly distributed in the square area. Each warden is paired with one cooperative jammer. The jammer is placed at a fixed distance $d_{J_{i},W_{i}}=2$ from its corresponding warden. The maximum single-hop transmission distance is 15. The ground-link transmit power, path-loss exponent, unit jamming cost, and utility coefficient are set to P=1, α=2, c=1, and λ=100. For each candidate base station, the ground utility $U_{B}$ is obtained from the optimized ground-route incentive mechanism. It is then used to compute the joint utility $U_{T}$.

For the satellite-to-ground segment, the LEO satellite transmit power is $P_{L}=10$ W. The main-lobe antenna gains of the LEO satellite and base station are both 40 dBi. The default sidelobe gain is −20 dBi. Two satellite layers with altitudes 1110 km and 1325 km are considered. The Rayleigh scale parameter is set to −12 dBi. Both the detection and outage thresholds are set to 0.1.

**Figure 5:**
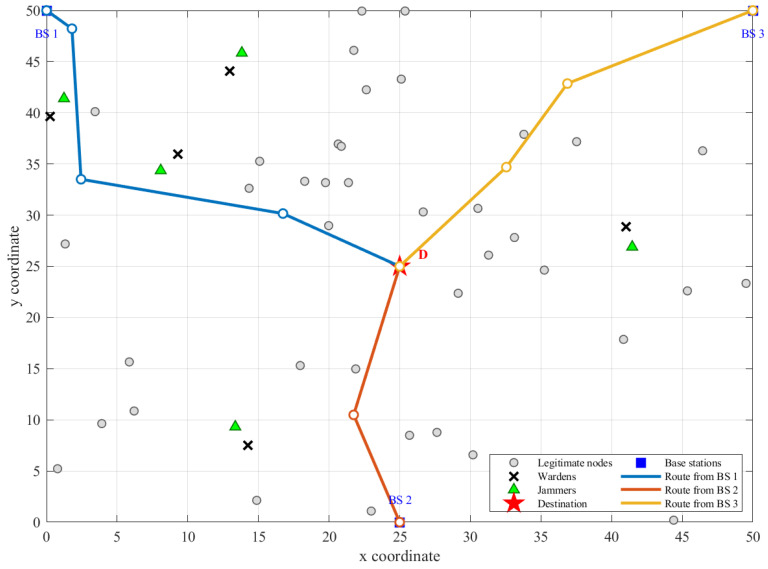
End-to-end route selection from candidate base stations to the destination.

Figure 5 shows a snapshot of the joint satellite-ground routing process. For each candidate base station, the ground routing module computes a covert multi-hop path to the destination. The satellite transmitter then evaluates the end-to-end utility using both the satellite-to-ground covert capacity $C_{L}$ and the ground utility $U_{B}$. The detailed results are listed in Table 2.

BS 3 achieves the highest end-to-end utility, although its ground utility is slightly lower than that of BS 2. The reason is that BS 3 provides a much larger satellite-to-ground covert capacity. This capacity gain compensates for its small loss in ground-route utility. This result illustrates why the final relay base station should be selected according to the joint metric $U_{T}$, rather than according to the ground route alone. Therefore, BS 3 is selected as the relay base station in this topology.

### 6.2. Parameter Sensitivity and Baseline Comparison

**Figure 6:**
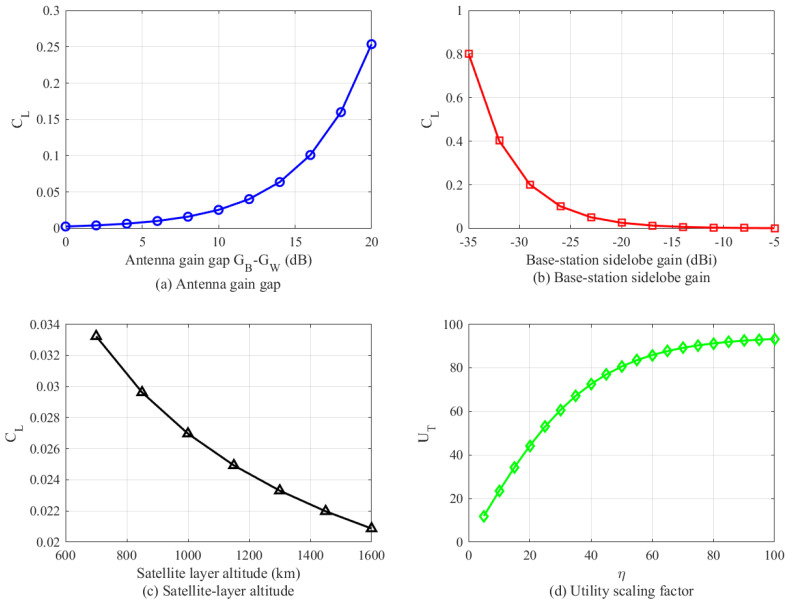
Impact of satellite-to-ground link parameters on covert capacity and end-to-end utility: (**a**) antenna gain gap; (**b**) base-station sidelobe gain; (**c**) satellite-layer altitude; (**d**) utility scaling factor.

Figure 6 evaluates how key satellite-to-ground link parameters affect the covert capacity and the end-to-end utility. In Figure 6a, the covert capacity $C_{L}$ increases as the antenna gain gap $G_{B}-G_{W}$ becomes larger. This is because a larger gain gap strengthens the reception advantage of the legitimate base station over the warden, thereby improving the achievable covert capacity. Figure 6b shows that increasing the base-station sidelobe gain reduces $C_{L}$. This is because stronger sidelobe reception increases the effective interference term under the considered approximation, which degrades the satellite-to-ground covert capacity. Figure 6c shows the effect of the satellite-layer altitude. As the satellite altitude increases, the propagation distance becomes larger, leading to a reduced covert capacity. Finally, Figure 6d presents the influence of the utility scaling factor η. The end-to-end utility $U_{T}$ increases with η at first and then gradually approaches saturation. This trend is consistent with the bounded property of the hyperbolic tangent function used in the joint utility formulation.

**Figure 7:**
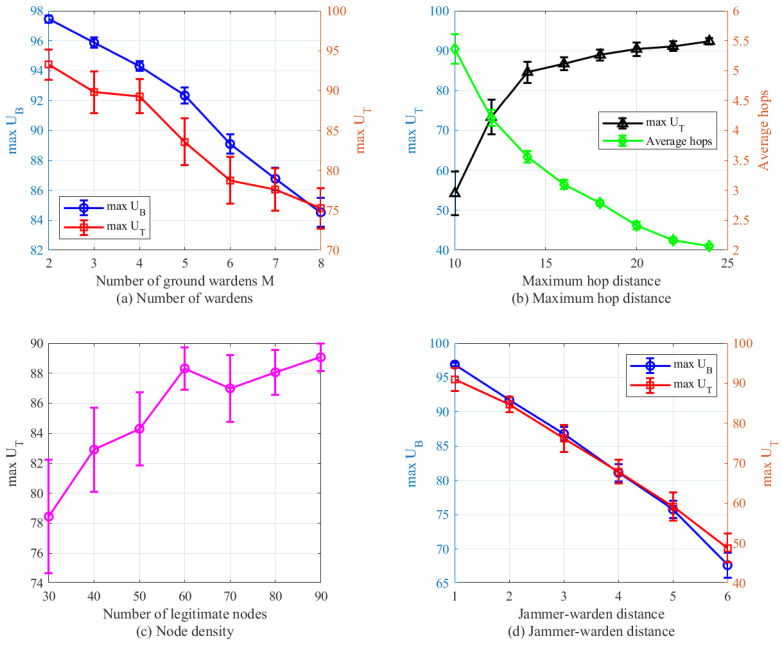
Impact of ground-network parameters on end-to-end performance: (**a**) number of wardens; (**b**) maximum hop distance; (**c**) node density; (**d**) jammer-warden distance.

Figure 7 investigates the impact of ground-network parameters on the proposed framework. Each point is averaged over 100 random network topologies. Figure 7a shows that both the maximum ground utility $U_{B}$ and the maximum end-to-end utility $U_{T}$ decrease as the number of ground wardens increases. This is expected because more wardens impose stronger monitoring pressure on the ground multi-hop route, which increases the cost of maintaining covert communication. Figure 7b shows the impact of the maximum single-hop transmission distance. A larger hop distance provides more feasible links for route construction and usually reduces the average hop count. As a result, the routing module has greater flexibility to bypass wardens and reduce the accumulated route weight, leading to a higher end-to-end utility. Figure 7c further shows that increasing the number of legitimate relay nodes improves the achievable end-to-end utility. This is because a denser relay deployment provides more candidate forwarding nodes, which enhances the route-selection flexibility. Under the considered node-density settings, the route success ratio remains one. Figure 7d demonstrates that a larger jammer-warden distance weakens cooperative jamming, since the artificial jamming signal becomes less effective at the warden. Therefore, both $U_{B}$ and $U_{T}$ decrease as the jammer-warden distance increases.

**Figure 8:**
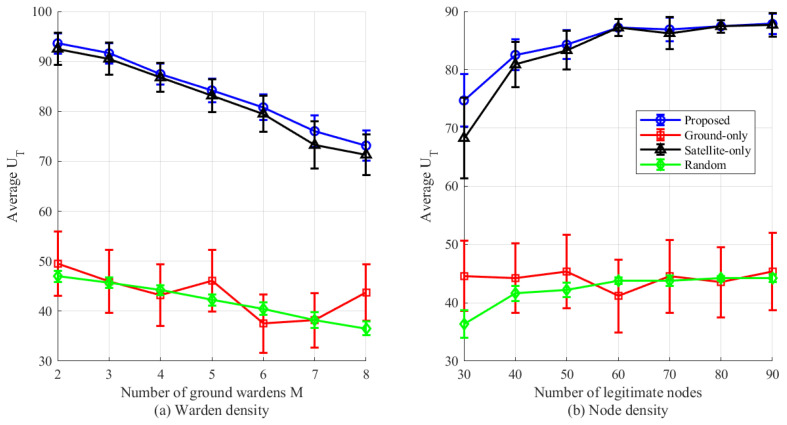
Comparison with baseline base-station selection schemes: (**a**) average $U_{T}$ versus number of wardens; (**b**) average $U_{T}$ versus number of legitimate nodes.

To demonstrate the necessity of joint satellite-ground selection, we compare the proposed scheme with three baseline strategies. The ground-only scheme selects the base station with the largest $U_{B}$, while the satellite-only scheme selects the base station with the largest $C_{L}$. The random scheme uniformly selects one candidate base station. If the selected base station does not have a feasible ground route in a given random topology, its end-to-end utility is counted as zero for that trial. It should be noted that Figure 8 is intended to compare different base-station selection strategies. After a candidate base station is selected, all the compared schemes employ the same incentive-aware ground-routing procedure, so that the observed performance differences arise specifically from the base-station selection criterion. All curves in Figure 8 are averaged over 100 random topologies, and the error bars represent the 95% confidence intervals.

Figure 8a compares the average end-to-end utility under different numbers of ground wardens. As the number of wardens increases, the average utility of all schemes decreases due to the increased monitoring pressure. Nevertheless, the proposed joint selection scheme consistently achieves the highest average $U_{T}$. The satellite-only scheme performs relatively close to the proposed scheme because $C_{L}$ plays an important role in the joint utility. However, since it ignores the quality and feasibility of the ground route, it may select a base station with weak ground forwarding performance. The ground-only scheme performs much worse because it neglects the satellite-to-ground covert capacity. Figure 8b shows a similar comparison when the number of legitimate relay nodes varies. These results verify that both the satellite-to-ground covert capacity $C_{L}$ and the ground utility $U_{B}$ should be jointly considered in relay base-station selection.

### 6.3. Long-Term Performance of the ZD Strategy

**Figure 9:**
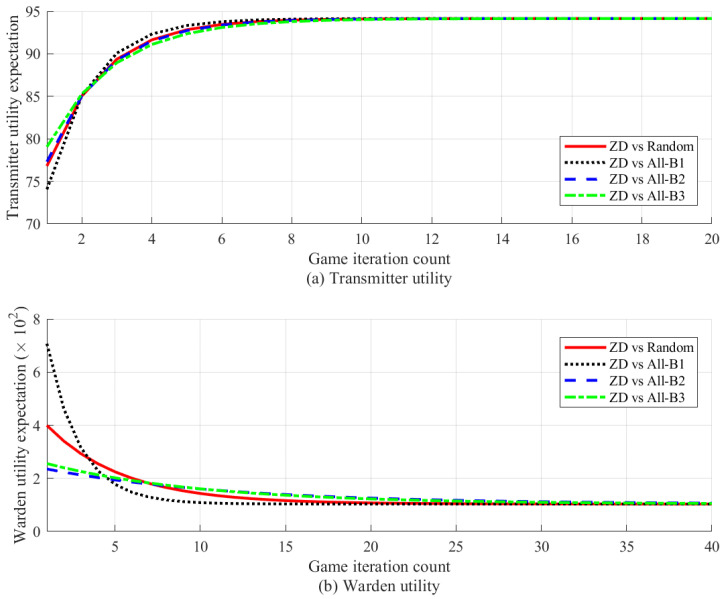
Expected utility convergence under the ZD strategy with different warden monitoring strategies: (**a**) transmitter utility; (**b**) warden utility scaled by $10^{2}$.

We further evaluate the long-term base-station selection process under the proposed ZD strategy. Four warden monitoring strategies are considered, namely random monitoring and fixed monitoring of BS 1, BS 2, or BS 3. The payoff matrix used in this experiment satisfies the ZD feasibility condition. Specifically, the target base-station row yields a higher transmitter payoff and a lower warden payoff than the non-target rows, which ensures that the constructed ZD probability vector remains within [0,1]. The simulation runs for 100 iterations. Since the utility trajectories converge rapidly, Figure 9a only shows the first 20 iterations of the satellite transmitter utility, while Figure 9b shows the first 40 iterations of the warden utility. The omitted iterations remain at the corresponding steady-state levels. For readability, the warden utility in Figure 9b is scaled by $10^{2}$.

As shown in Figure 9, different warden monitoring strategies result in different transient utility trajectories. However, under the ZD strategy, the long-term expected utilities converge to strategy-independent target values. In particular, the transmitter-side utility quickly approaches its stable level, while the warden-side utility also converges within the displayed iterations. This confirms that the ZD strategy can stabilize the long-term interaction between the satellite transmitter and the warden under different monitoring behaviors.

**Figure 10:**
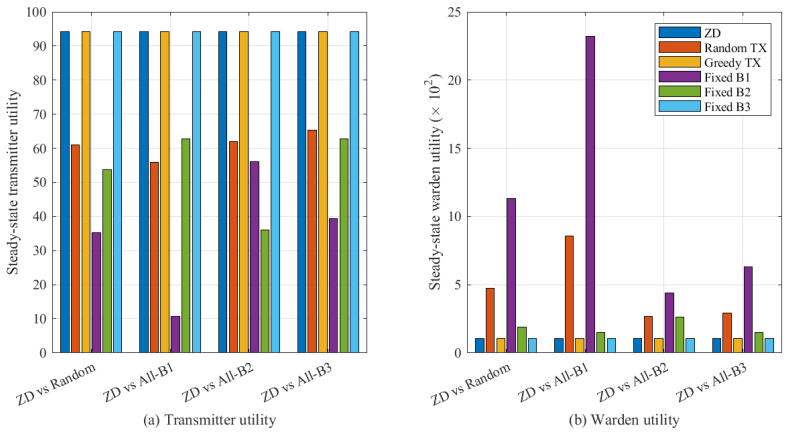
Steady-state performance comparison between ZD and non-ZD transmitter policies: (**a**) transmitter utility; (**b**) warden utility scaled by $10^{2}$.

Finally, we compare the steady-state performance of the ZD strategy with several non-ZD transmitter policies, including random transmitter selection, greedy transmitter selection, and fixed selection of BS 1, BS 2, or BS 3. The steady-state utility of the ZD strategy is calculated from the last 10 iterations of the 100-iteration simulation. As shown in Figure 10, the ZD strategy reaches the target-level transmitter utility under all considered warden monitoring policies and maintains the warden utility at a low level. Greedy selection and fixed BS 3 can achieve similar transmitter utility in this payoff matrix because BS 3 is the dominant target base station. However, unlike the ZD strategy, they cannot provide a payoff-pinning mechanism that stabilizes the long-term expected utility relation. Random selection and non-target fixed strategies are more sensitive to the warden monitoring policy, resulting in either lower transmitter utility or higher warden utility. These observations demonstrate the advantage of the ZD strategy in achieving stable long-term base-station selection under uncertain warden behavior.

## 7. Conclusions

In this paper, we investigated the problem of incentive-aware end-to-end covert routing in space–air–ground integrated networks. We considered a two-stage communication process in which a LEO satellite forwards information to a ground destination through a selected relay base station and a self-organizing multi-hop ground route. To characterize the end-to-end covert performance, we first analyzed the satellite-to-ground covert capacity under satellite sidelobe interference and evaluated the ground-route covertness in the presence of multiple wardens and cooperative jammers. Since the jammers are self-interested and incur power costs when generating artificial interference, we designed an incentive mechanism to encourage cooperative jamming while accounting for both the covertness gain and the jamming-power cost. Based on the derived route-dependent utility, the ground routing problem was further transformed into a shortest-weighted path-finding problem, enabling efficient route construction. Moreover, to improve the long-term stability of satellite-to-ground relay access, we introduced a zero-determinant strategy for the repeated base-station selection process between the LEO satellite transmitter and the satellite warden. Simulation results demonstrated that the proposed framework can effectively balance satellite-to-ground covert capacity and ground-route utility, outperform baseline selection schemes, and stabilize long-term expected utilities under different warden monitoring strategies.

Future work will extend the proposed framework to more practical SAGIN scenarios characterized by uncertain warden locations, warden mobility, limited availability of cooperative jammers, and delayed or imperfect channel information, to develop robust and adaptive routing and jammer-selection mechanisms. We will also consider adaptive adversarial settings in which wardens exploit historical interactions, estimate the transmitter’s selection policy, or learn best-response monitoring strategies. Furthermore, packet-level traffic, transmission duration, and explicit detection models will be incorporated to enable a more fine-grained evaluation of false-alarm and missed-detection probabilities, outage probability, covert throughput, route length, end-to-end delay, total jamming energy, and incentive expenditure.

## Figures and Tables

**Table 1:** Main simulation parameters.

Parameter	Value
Network region	50×50
Utility coefficient λ	100
Number of legitimate relay nodes	49
Destination coordinate	(25,25)
Candidate base-station coordinates	(0,50), (25,0), (50,50)
Number of ground wardens *M*	5
Jammer-warden distance $d_{J_{i},W_{i}}$	2
Maximum hop distance	15
Ground transmit power *P*	1 W
Number of satellite layers $N_{r}$	2
Total number of satellites *N*	600 (300 per layer)
System bandwidth	20 MHz
Noise power spectral density	−174 dBm/Hz
Receiver noise power	−94 dBm
Path-loss exponent α	2
Unit jamming cost *c*	1
Satellite transmit power $P_{L}$	10 W
Main-lobe antenna gains	40 dBi
Default sidelobe gain	−20 dBi
Satellite-layer altitudes	1110 km, 1325 km
Detection/outage thresholds	0.1/0.1
Joint-utility scaling factor η	50
Number of random topologies	100
Number of ZD iterations	100

**Table 2:** End-to-end utility of candidate base stations.

Base Station	Coordinate	Hops	Δ	$R^{*}$	$D_{e}$	$U_{B}$	$C_{L}$	$U_{T}$
BS 1	(0,50)	4	3.8174	9.9802	0.0293	84.3276	0.0025	10.6380
BS 2	(25,0)	2	0.7746	3.6474	0.0099	94.3926	0.0080	35.9500
BS 3	(50,50)	3	0.8259	3.8004	0.0103	94.1540	0.2537	94.1540

## Data Availability

The source code utilized in this study is available upon reasonable request from the corresponding author.
